# Heritable DNA Methylation in CD4^+^ Cells among Complex Families Displays Genetic and Non-Genetic Effects

**DOI:** 10.1371/journal.pone.0165488

**Published:** 2016-10-28

**Authors:** Kenneth Day, Lindsay L. Waite, Arnald Alonso, Marguerite R. Irvin, Degui Zhi, Krista S. Thibeault, Stella Aslibekyan, Bertha Hidalgo, Ingrid B. Borecki, Jose M. Ordovas, Donna K. Arnett, Hemant K. Tiwari, Devin M. Absher

**Affiliations:** 1 HudsonAlpha Institute for Biotechnology, Huntsville, Alabama, United States of America; 2 University of Alabama at Birmingham, School of Public Health, Department of Biostatistics, Birmingham, Alabama, United States of America; 3 University of Alabama at Birmingham, School of Public Health, Department of Epidemiology, Birmingham, Alabama, United States of America; 4 Washington University, Division of Statistical Genomics, St. Louis, Missouri, United States of America; 5 Tufts University, Jean Mayer USDA Human Nutrition Research Center on Aging, Boston, Massachusetts, United States of America; 6 IMDEA-Food, Madrid, Spain; RWTH Aachen University Medical School, GERMANY

## Abstract

DNA methylation at CpG sites is both heritable and influenced by environment, but the relative contributions of each to DNA methylation levels are unclear. We conducted a heritability analysis of CpG methylation in human CD4^+^ cells across 975 individuals from 163 families in the Genetics of Lipid-lowering Drugs and Diet Network (GOLDN). Based on a broad-sense heritability (*H*^*2*^) value threshold of 0.4, we identified 20,575 highly heritable CpGs among the 174,445 most variable autosomal CpGs (SD > 0.02). Tests for associations of heritable CpGs with genotype at 2,145,360 SNPs using 717 of 975 individuals showed that ~74% were cis-meQTLs (< 1 Mb away from the CpG), 6% of CpGs exhibited trans-meQTL associations (>1 Mb away from the CpG or located on a different chromosome), and 20% of CpGs showed no strong significant associations with genotype (based on a p-value threshold of 1e-7). Genes proximal to the genotype independent heritable CpGs were enriched for functional terms related to regulation of T cell activation. These CpGs were also among those that distinguished T cells from other blood cell lineages. Compared to genes proximal to meQTL-associated heritable CpGs, genotype independent heritable CpGs were moderately enriched in the same genomic regions that escape erasure during primordial germ cell development and could carry potential for generational transmission.

## Introduction

DNA methylation is an epigenetic mark found within the context of CpG dinucleotides that modifies gene expression in a cell type-specific manner, and is deposited or removed particularly during cellular differentiation and development.

DNA methylation changes are also strongly associated with environmental stimuli, such as diet or smoking, which may contribute to disease in a reversible manner [1–4]. Some evidence has emerged that these environmentally-induced epigenetic effects may be transmitted to the next generation in mammals, but whether methylation marks persist in the germline is still under intense investigation [5–7]. DNA methylation within the mammalian genome is erased and reprogrammed during embryogenesis, including primordial germ cells, in which epigenetic mark erasure has the potential to influence future generations [8]. Recent genome-wide methylation studies in sorted human primordial germ cells at different developmental phases indicates that some DNA methylation escapes erasure and may represent sites with potential for generational transmission and disease association [9].

It is unclear how much human epigenetic variation is heritable and to what degree that heritability is dependent on genotype. Sequence-independent heritable methylation has been well documented in plants [10–13], and some of these effects have also been observed in mammalian species including mice [14–16]. There is little understood about sequence-independent heritable DNA methylation in humans, but some evidence has emerged [17,18]. Many studies have demonstrated the association between CpG methylation and genotype at specific single nucleotide polymorphisms (SNPs), also known as meQTLs or allele-specific methylation [19–26]. Additionally, studies of twins have revealed interesting results concerning the stability of heritable DNA methylation states, genotype, and the influence of the environment. Analyses of monozygotic and dizygotic twins have demonstrated that monozygotic twins have much stronger correlations in methylation than dizygotic twins, and comparison of these twin types showed that much of the discordance in methylation is independent of genotype [27–30]. Recent heritability studies in a large cohort of 2603 individuals from monozygotic and dizyogotic twins using classical twin model approaches estimated that unique environmental effects explained 80% of variance in DNA methylation in whole blood, with very little contribution of shared environmental effects [31]. Results from a smaller twin cohort corroborated this, showing no significant shared environment effects [32]. Analysis of blood samples across the human lifespan showed stability in the genetic contribution to variation in methylation, and developmentally-related changes in this genetic contribution were explained by increased environmental effects [33]. This stability of methylation therefore appears to be the result of both environmental and genetic constraints [34]. Generally, studies of monozygotic twins have shown that DNA methylation profiles are practically identical at birth, but diverge with increasing age [27,30,35].

The heritability of DNA methylation among families has not been comprehensively described, and family-based studies offer potential for disentangling the sequence effects on methylation patterns. We conducted this study to better understand and characterize the strongest heritable DNA methylation among complex, extended families using Illumina HumanMethylation450 array data from CD4^+^ cells among families enrolled in the Genetics of Lipid-Lowering Drugs and Diet Network (GOLDN) study [36,37]. We measured the broad-sense heritability (*H*^*2*^) at each CpG and assessed how many of the CpGs exhibiting the highest heritability could be explained by SNP associations (meQTLs) annotated by their distance to the CpG (less than or greater than 1 Mb). We also examined the heritability patterns of these CpGs by examining the association between heritable methylation for different types of familial relationships, and compared patterns in SNP-associated meQTLs with those CpGs that did not show a significant genetic association (genotype independent CpGs; GICs). We further found that heritable methylation patterns at GICs is enriched near genes related to T cell activation. Altogether, our study represents the largest cohort of complex extended families for which heritable DNA methylation was determined in a sorted cell type, and demonstrates that some heritable methylation across families cannot be explained by genotype.

## Results

**Fig 1:**
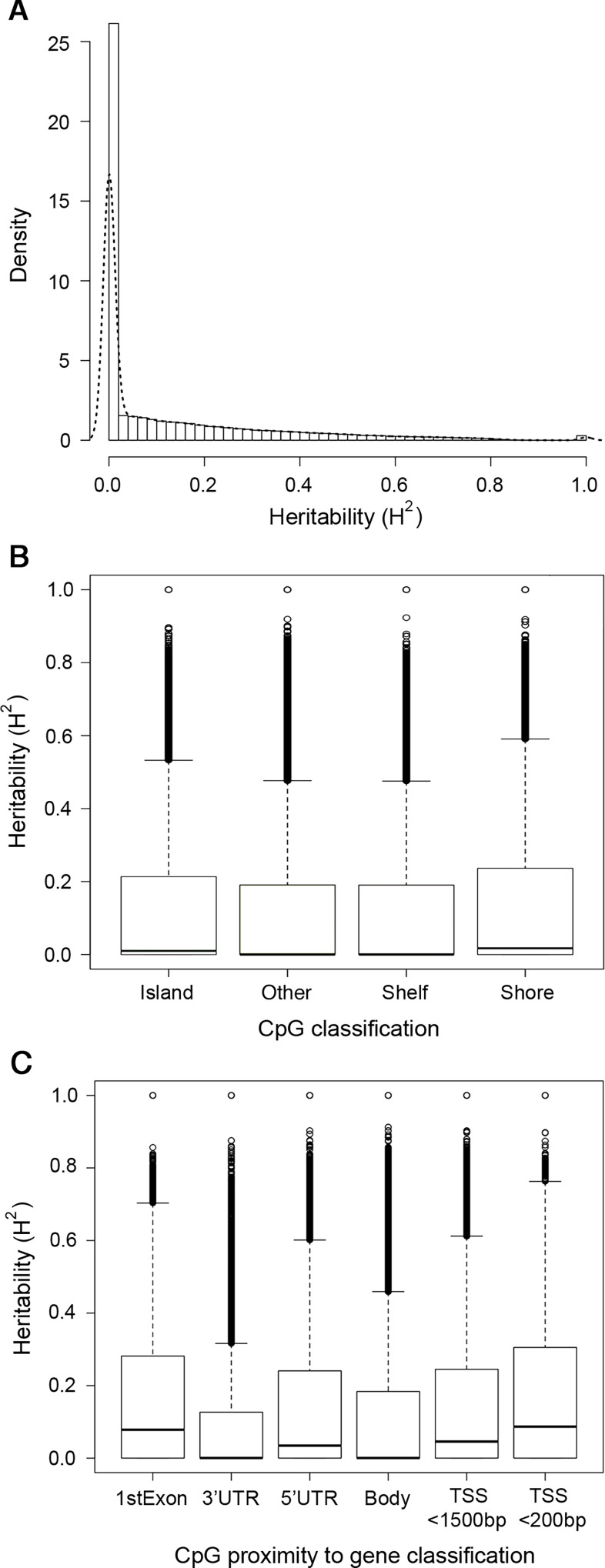
Distributions of heritability values by CpG island proximity and gene region. Histogram of heritability values (A) for all 174,445 tested CpGs with overlaid density estimate (dotted-line) and boxplots of heritability values for CpGs annotated by CpG-island proximity (B), and gene region (C).

Sorted CD4^+^ cells were isolated from 975 individuals and methylation data were collected from Illumina methylation450 arrays following batch normalization and quality control. Heritability estimates were obtained across 174,445 of the most variable CpGs assayed on the methylation450 array with standard deviations above 0.02. Approximately 64% of CpGs produced heritability estimates less than 0.1, and the average *H*^*2*^ value was 0.1312 (Fig 1A, S1 Table). Among all genomic regions, heritability values were the highest for methylation sites located within CpG shores (Fig 1B). Heritability values also were generally larger for CpGs located in first exon and promoter regions of genes (5’ UTR, TSS<200bp and TSS<1500bp), compared to CpGs located within the gene body or 3’UTR (Fig 1C).

### SNP Association with Heritable CpGs

A heritability threshold of *H*^*2*^>0.4 was used to produce a list of 20,575 highly heritable CpGs. These CpGs were tested against 2,145,360 SNPs to determine associations between methylation and genotype (meQTLs). The tests were first conducted within a 5 kb window, resulting in significant associations (p<1e-7) for 11,915 of 20,575 heritable CpGs. For any remaining heritable CpGs, the window was extended to a 20 kb distance (1,551 additional CpGs), then 100 kb (1,018 CpGs), 1 Mb (649 CpGs), 3 Mb (80 CpGs), the whole chromosome (198 CpGs), and finally the entire genome (1,051 CpGs). Of the 20,575 heritable CpGs, 4,113 CpGs showed no significant association for any SNP across the genome.

**Fig 2:**
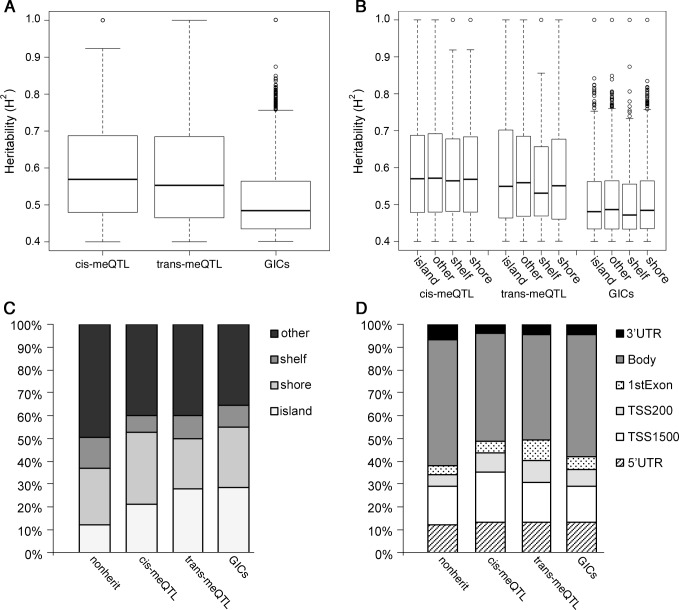
Distributions of heritability values across heritable CpGs by SNP-association group, CpG island proximity, and gene region. Boxplots show heritability values for heritable CpGs under our threshold within their respective SNP-association groups (A). Boxplots of heritability values were also plotted for each SNP-association group subdivided by CpG island proximity (B). Barplots depict relative percentages of heritable CpGs across SNP-association groups that were positioned within CpG island proximity (C) or gene region (D) annotations.

Highly heritable CpGs were categorized by SNP-association into the categories of cis-meQTLs (15,133 CpGs with associated SNP ≤ 1 Mb from CpG), trans-meQTLs (1,329 CpGs with associated SNP > 1 Mb from CpG), and genotype independent CpGs (GICs; 4,113 CpGs with no SNP association meeting the threshold of p<1e-7). Heritability values were generally largest for cis-meQTLs and smallest for GICs (Fig 2A). There was also less variation in heritability values for CpGs in the GIC category. Relative positions by CpG island location showed very little differences among heritability values for cis-meQTLs, but trans-meQTLs and GICs had more differences among CpG annotations, with CpG shelves displaying the lowest heritability values (Fig 2B). Trans-meQTL and GICs were enriched in CpG islands compared to the cis-meQTL category (Fig 2C; Pearson’s Chi-Square Test, p<2e-16). Location of highly heritable CpGs by gene region demonstrated that a larger proportion of meQTL heritable CpGs (cis and trans) were located in the promoter (5’UTR, TSS1500, and TSS200) and first exon regions of genes while GICs and low or non-heritable CpGs (*H*^*2*^<0.4) were found in larger proportions within the gene body and 3’UTR (Fig 2D; Pearson’s Chi-Square Test, p< 2e-16). Highly heritable CpGs were also annotated within chromatin states across different cell lineages by Roadmap epigenomics data (S1 Fig). Highly heritable CpGs (*H*^*2*^>0.4) were enriched in chromatin states associated with active transcriptional start sites (E1 and E2) in 15-state models compared to low or non-heritable CpGs (*H*^*2*^<0.4) across many cell types with ES cell types showing the strongest odds ratios (S2 Fig). We did not observe any strong separation in chromatin states according to their associations with SNPs.

### Family correlation estimates for relative pairs

**Fig 3:**
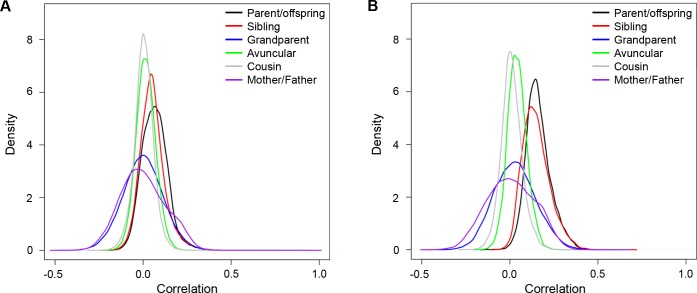
Estimated kernel density plots of family correlations for the main family relationship types. Density plots for relationship types are parent-offspring (539 pairs), sibling-sibling (588 pairs), grandparent-grandchild (87 pairs), avuncular (aunt/uncle-niece/nephew) (782 pairs), cousin-cousin (600 pairs), and mother-father (husband-wife) (86 pairs). Densities are plotted for all 170,915 evaluated CpGs (A) and 20,163 heritable CpGs (B).

We further estimated family correlations for 170,915 of the 174,445 tested CpGs. Correlation values for each relationship pair revealed that familial correlations among all tested CpGs for all relationship types had a median close to zero, corresponding to our previous observation that most CpGs were not highly heritable (Fig 3A). Among highly heritable CpGs, parent-offspring and sibling-sibling correlation values generally exceeded correlations between more distant relative pairs (grandparent-grandchild, avuncular, and cousin-cousin), as would be expected under a genetic inheritance model (Fig 3B and S2 Table). Correlation estimates were generally higher among cis-meQTL heritable CpGs than trans-meQTL CpGs, which were together higher than heritable GICs (S2 Table). These results were expected given the distribution of heritability estimates in these classes of CpGs (Fig 2A). Family correlation values were also estimated for gender-specific subtypes of relatives. Few noticeable differences in correlation distributions were found between gender-specific subsets of relatives either for the entire set of 170,915 tested CpGs or for the subset of 20,163 heritable CpGs (S3 Fig and S2 Table). However, correlations for pairs of the same gender were slightly higher than for pairs of different gender.

It was possible that GIC heritable methylation could be driven by rare variants that only occurred in one or two families. These rare variants would not be detected among associations with common SNPs throughout the genome. We considered that if heritability of GICs was driven by rare variants in a few families, then we would expect that many of the most extreme maximum or minimum beta scores at some CpGs would have several individuals overrepresented from a single family. We tested this hypothesis by selecting the top ten maximum and minimum beta scores for each GIC. We observed that 3795 of 4113 GICs had 9 or 10 families represented within the maximum beta scores, and 3,674 of these 4,113 CpGs had 9 or 10 families represented in the minimum values. Additionally, 4,019 of 4,113 CpGs had no greater than 2 individuals per family represented in the maximum beta scores, and 3,990 of 4,113 CpGs in the minimum values. Only 28 CpGs had more than 3 individuals from a single family represented in the top 10 most extreme beta scores. This test did not completely eliminate the possibility that some rare variants may be associated with some GICs, but showed that a small number of families do not appear to drive the majority of heritable methylation among GICs.

### Functional annotation analysis of GIC heritable CpGs

**Fig 4:**
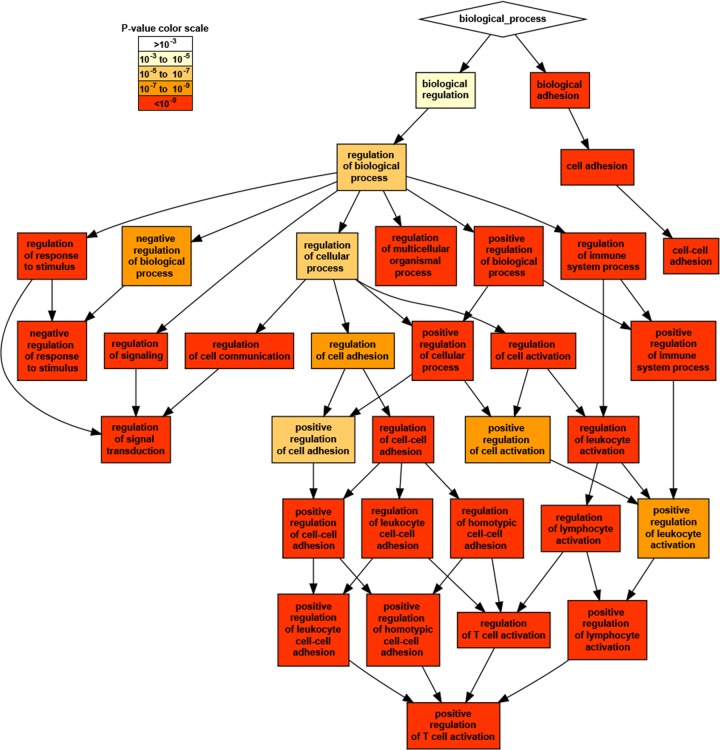
GO-term enrichments for GICs. Directed acyclic graph created by GOrilla using Graphviz, displaying the most significantly enriched GO-process annotations for the annotated genes near GICs.

A gene ontology (GO) term analysis was conducted for the genes proximal to heritable CpGs according to cis-meQTL, trans-meQTL, and GIC classifications. There were few strongly specific GO term enrichments found for the set of genes proximal to cis-meQTL heritable CpGs (biological adhesion and cell-cell adhesion, p<1e-10), and trans-meQTL CpGs. However, genes proximal to GICs produced strongly significant enrichments in GO annotation terms of immune process, lymphocyte activation, and the most specific term was positive regulation of T cell activation (Fig 4).

We were further interested in whether GICs were associated with genotype in other studies. We compared our heritable CpGs with reported meQTLs in primary T cells [38] and found overlap with ~62%, 7%, and 16% of our cis-meQTLs, trans-meQTLs, and GICs, respectively. We depleted our GIC list of these T cell specific meQTLs and other independently reported meQTLs from whole blood [33]. Another ~34% of GICs were removed, but the GO enrichment for regulation of T cell lymphocyte activation remained across associated genes (3.6 fold, p = 7.5e-8, FDR = 1.4e-4). Any GICs proximal to the same genes near cis- or trans-meQTLs were also removed, and the enrichment in T cell activation term persisted (4.5 fold, p = 2.7e-6, FDR = 0.005). More GIC-related genes were shared with trans-meQTL-related genes than cis-meQTLs (14% and 9.5% overlap, respectively, Fisher test, p = 0.0002). We further filtered this top GIC list to greater than 2 GICs within 10 kb distance from one another (S3 Table). Approximately 22% of these GICs were located around the MHC region annotated near genes such as PRRT1, B3GALT4, KIAA1949, and BAT4. Depletion of MHC locus-related genes from the full GIC list (depleted of meQTL and meQTL-related genes) also did not remove GO term enrichment for T cell activation. One GIC region was the PRSS50 promoter, which was shown to be associated with age-related macular degeneration in both blood and retina samples [39]. Another site was around the LTB4R gene that encodes the leukotriene B4 receptor, and was shown to be the most epigenetically divergent human gene compared to other primates [40].

### Mixed effects among GIC heritable CpGs

Based on the robust signal of GICs in genes involved in regulation of T cell activation, we found that many of the genes driving this signal were involved in T cell signaling such as CD3e, CD28, CD80, and CD247. We were interested in whether GICs (depleted of meQTLs from other studies) showed any differences in methylation status during T cell differentiation. We used methylation array data from our recently reported blood cell estimation study and isolated strong methylation differences (+/-25%) among sorted blood cell beta scores, including differentiated T cells from naïve T cells [41,42]. Some GICs overlapped (142/2261) with these differences common among all differentiated T cell types (173, 208, and 227 GICs overlapped specific to Th1, Th2, and Th17 cells, respectively). However, genes proximal to these GICs were not associated with the regulation of T cell activation GO term enrichment. We found that the greatest variation among GIC methylation was actually between T cells and non-T cells (monocytes, granulocytes, NK, and B cells; S4 Fig). Hierarchical clustering of GIC-associated beta scores from sorted cell types showed two main clades that partitioned all T cells from non-T cells (S4 Fig). The greatest numbers of cell differences among GICs (+/-50%) were between T helper and monocyte or granulocyte lineages with no strong differences among T cell populations (S5 Fig). Genes associated with these cell-difference associated GICs produced strong GO enrichment in regulation of T cell activation (6.5 fold, p = 6.9e-8, FDR = 1.2e-4). Furthermore, GICs were much greater enriched in strong cell-difference associated CpGs compared to heritable meQTLs (Chi square test, p = 3e-54). Approximately 94%, 85%, and 70% of monocyte-, B cell-, and NK cell- differences from T helper cells in GICs overlapped with granulocyte-difference related CpGs, respectively.

Monocytes and granulocytes are known to express CD4 [43,44]. Among individual cell estimates from beta scores using our cell estimation model, CD4^+^ T cells and granulocytes represented the largest percent variation and greatest relative percentages overall (median of 62% and 20%, respectively) [41]. CD4^+^ T cells and granulocyte estimates were both strongly anti-correlated to each other and each strongly correlated with principal component 1 included in our mixed models (83% and 94% variance explained, respectively, S6 Fig). Analysis of principal components on residuals from our original heritability models did not show significant associations of granulocyte cell estimates with residual variation. Although monocytes showed a strikingly similar pattern to granulocytes within cell-difference associated GICs, they represented a minor fraction among all of our cell estimates (median of 1.4%) in addition to B and NK cells (median of 0.2%, and 0%, respectively). Therefore, our T cell activation-related gene enrichment in GICs is not due to a lack of cell type correction in our heritability models.

**Fig 5:**
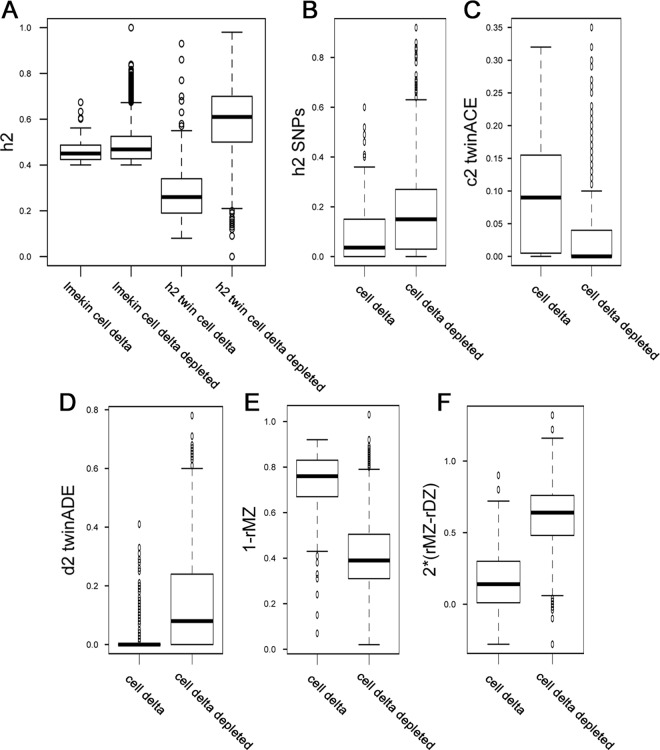
Boxplots of twin data for strong cell-difference related GICs and non-cell difference related GICs. Boxplots of H2/h2 values across our cell-difference related (cell delta) or non-cell type difference related (cell delta depleted) with respect to our H^2^ values (lmekin) and twin data (h2 twin) (A). Boxplots depicting twin data values within cell delta or cell delta depleted GICs: h2 values associated with genotype (h2 SNPs; B), common environmental values (c2 twinACE;C), non additive genetic effects (d2 twin ADE;D), unique environmental values (1-rMZ; E), and additive genetic effects (2*rMZ-rDz; F).

Some heritable methylation within GICs could be attributed to environmental influences. Twin studies uniquely allow estimation of environmental versus genetic influences on DNA methylation [45]. We used methylation450 data from a recent large cohort classical twin study in blood to further annotate our heritable CpG categories [31]. We first compared our heritability estimates to twin heritability estimates across our 170,915 tested CpGs and found them to be well correlated (R^2^ = 0.58), but our estimates were generally lower with a slope of 0.66. We also set our heritability threshold in the twin data (h^2^twinAE > 0.4) and found that 77% of our low or non-heritable CpGs (H^2^ <0.4) were also low or non-heritable in twin data, and 95%, 83%, and 87% of our highly heritable cis-meQTL, trans-meQTL, and GICs overlapped under this threshold (H^2^>0.4). These data indicated that heritable methylation among families within sorted CD4^+^ cells corroborated with twin data using whole blood. Twin methylation heritability values in CpGs associated with SNPs also agreed with our data among our heritable CpG categories, and no large differences were found among our categories in measurements such as common environment values (c^2^) and non-additive genetic values (d^2^) (S7 Fig). Also similar to our results, GICs showed the lowest heritability in twin data. GICs also showed the greatest unique environment values among our heritable CpGs (1-rMZ, S7 Fig). Separation of GICs by strong differences in methylation (+/- 50%) between T cells and granulocytes further showed that strong cell-difference related GICs held much higher values for common and unique environment effects with correspondingly lower values for genetic effects in twin data (Fig 5). These data suggest a mixture of effects among our heritable GICs, where some of the heritable effect could be attributed to environmental influence.

We were also interested in potential epigenetic generational effects related to environment within GICs. We tested for overlap of genes associated with GICs within 25kb of unique sites in the genome that escape methylation erasure (escapee methylation) as determined by Tang et al. during primordial germ cell development [9]. We found no enrichment in cell difference-related GICs compared to cell-difference depleted GICs, but GICs altogether were moderately enriched in genes near escapee methylation sites compared to heritable meQTLs (Fisher’s test, p = 0.008). Heritable CpGs (meQTLs and GICs together) compared to low or non-heritable CpGs (H^2^ <0.4) were highly enriched in genes near escapee methylation sites (20% and 13.4%, respectively, p<2e-16). Metastable epialleles (MEs) are also potential candidate sites for environmentally-driven methylation patterns established during embryogenesis that differ among individuals and persist within differentiated tissues [46–48]. We tested for enrichment of our heritable CpGs within 25kb of MEs as determined by Silver *et al*. using whole genome bisulfite sequencing [46]. There was overall less overlap with MEs compared to escapee sites (heritable CpGs, 5.4%; low or non-heritable CpGs, 4.3%), with significant enrichment of heritable CpGs near MEs (p = 2e-10). Interestingly, there was greater enrichment of heritable meQTLs near ME sites compared to GICs (5.7% and 3%, respectively, p = 5.304e-08), with trans-meQTLs showing slightly more enrichment in MEs than cis-meQTLs (7.2% and 5.5%, respectively, p = 0.01). We also compared overlap of 1776 ME CpGs determined by Harris *et al*. using HumanMethylation450 arrays [48] with our heritable CpGs and found small enrichment of meQTL CpGs within MEs compared to GICs (3.4% and 1.9%, respectively, p = 8.5e-05). Altogether, heritable CpGs according to array data were also greatly enriched in MEs compared to low or non-heritable CpGs (p = 2.5e-249). Compared to escapee sites, it appears that MEs are greater enriched in meQTL-related heritable methylation.

## Discussion

Our study is the first to investigate the heritability of DNA methylation in complex families among sorted CD4^+^ cells and associations with sequence variants in highly heritable CpGs. Most DNA methylation heritability studies were performed in twins and mean values were between 0.18 and 0.19 [31–33,49,50]. However, we estimated mean heritability at 0.13 similarly to a recent Mexican-American family study that reported a mean heritability value of 0.14 using whole blood samples [51]. Lower average heritability levels of DNA methylation have been observed in other tissues (0.12 in cord blood mononuclear cells, 0.07 in human umbilical vascular endothelial cells, and 0.05 in placenta) [52]. We also observed highest heritability in CpG shore regions across all tested CpGs, corroborating with another report of lower heritability in regions with high CpG density (CpG islands) compared to intermediate or low CpG density (CpG shores) [32]. Overall, highly heritable methylation is enriched in regions outside of CpG islands, and analysis of Roadmap data also showed enrichment of these CpGs in transcriptionally active chromatin regions, especially in ES cells.

Our strongest heritable methylation was associated with nearby sequence variants, and distant genetic variants (trans-meQTLs) have also been found in other studies [32,53,54]. While some heritable methylation is explained by genotype, we found a small number of CpGs that were not associated with SNPs after filtering for meQTLs identified across other studies in T cells and whole blood [33,38]. Another analysis in 22 nuclear pedigrees with 52 parent-trios also showed that meQTLs could not explain the full extent of heritable methylation [54]. Our findings showed that ~20% of highly heritable CpG methylation (*H*^*2*^>0.4) was not strongly associated with common sequence variants.

We found a functional GO enrichment of GIC-related genes in T cell activation terms. The corresponding GICs were enriched in sites showing strong methylation differences that distinguished CD4^+^ T cells from other non-T cells also reported to express CD4 to some degree. Based on our derived cell estimates from methylation in sorted CD4^+^ cells, the contribution of granulocytes was the second largest population after T helper cells. While this seemed to suggest potential heritability of CD4^+^ T cell to granulocyte ratios since the genes found near these GICs would presumably be expressed in all T cells (these genes are required for T cell activation and differentiation, and are hypomethylated relative to other cell types), our data showed that no remaining variation could be attributed to minor cell types according to cell estimates and principal components that should explain reported heritable cell type numbers [55–59]. Some of these heritable methylation effects could be environmentally driven by exposure to similar pathogens among family members [60]. Analysis of GICs in twin data showed that GICs related to strong cell differences between CD4^+^ T cells and granulocytes held much larger values for unique and shared environment compared to other GICs. It is also possible that for these strong cell delta-related CpGs, methylation signatures are mixed between CD4^+^ T cells and another CD4^+^ cell type, thus obscuring any association with genotype. However, with the specificity of these CpGs near genes required for T cell activation, it is also possible that heritable methylation changes may rather confer a specific regulatory role that suppresses T cell activation through increased methylation near genes such as CD3e, CD28, Thy-1, IL-21, IL4R, and IL7R, that were typically hypomethylated in CD4^+^ T cells. One recent study that investigated the reproducibility of individual T cell response showed that ~25% and ~4% of T cell activation could be explained by cis genetic effects and physiological covariates, respectively, and the rest reflected contributions from the environment or immunological history [61]. Therefore, it could be possible that altered methylation at key co-stimulatory genes could impact reproducibility in T helper cells. There is also evidence for a heritable component to V(D)J gene segment usage that could potentially influence similar T cell responses via T cell receptor repertoires among related individuals [62]. More sophisticated sorting schemes among the CD4^+^ cell population in future experiments may clarify this mixed effect among GICs.

It is not possible to completely rule out rare variants or other types of sequence variants such as indels or copy number variations that may be driving the heritability of GICs. Interestingly, 22% of our top GIC region list was located within around the MHC locus, and it is possible we have not detected rare alleles around MHC that may drive a cis genetic effect on heritable methylation. To the best of our knowledge, it is also unknown if there are trans-meQTL associations between genotype at the MHC locus and methylation on other chromosomes, and given our enrichment in T cell activation terms and the role of MHC in T cell response, this is of interest. Furthermore, the multiple testing burden imposed by testing such a large number of SNPs makes it difficult to rule out any true associations that may be below our significance threshold.

It is also equally difficult to rule out the environmental effects that may be driving associations among heritable methylation within GICs in families, but twin data showed GICs held the highest environmental values among other heritable CpGs categories. Interestingly, the positive correlation between married couples in GICs was larger than that for other categories of heritable CpGs, yet it is still much smaller than the parent-offspring and sibling-sibling correlations. This suggests that although the heritability observed in some of the GICs may be driven by shared environment, most of the heritability observed in the GICs cannot be explained by shared environment alone. Furthermore, the grandparent-grandchild correlation was sharply reduced in GICs, suggesting that some of these effects are not trans-generational. However, enrichment of genes among GICs associated with methylation near gene regions that escape erasure in primordial germ cells shows that conceivably some GIC heritable methylation could be generationally transmitted. Altogether, heritable methylation regardless of SNP association is enriched within escapee sites and MEs, but GICs were enriched within escapees compared to meQTLs, and the reverse effect was observed in MEs. Future work may be directed toward better understanding the function of GICs, and whether these CpGs confer similar gene expression levels among related individuals. Overall, there is a mixture of effects among CpGs that display heritable methylation patterns related to genotype, cell type, environment, and enrichment near genes that have potential to exhibit generational epigenetic effects.

## Materials and Methods

### Data collection and quality control

Data were obtained from buffy coat samples collected from participants at baseline (prior to high fat shake or fenofibrate treatments) in the Genetics of Lipid-Lowering Drugs and Diet Network (GOLDN) study [36,37]. Informed written consent was obtained from all participants, and the study was approved the Institutional Review Board at the University of Alabama at Birmingham (IRB Protocol #X040826013). In order to obtain DNA methylation measurements, DNA was isolated from CD4^+^ cells from previously frozen buffy coat samples using Invitrogen Dynabeads [63] for 975 individuals from 163 pedigrees, ranging in size from 2 members to 35 members. For each sample, 500 ng of DNA was extracted and treated with sodium bisulfite (Zymo EZ DNA). DNA methylation was quantified using Illumina Human Methylation 450K arrays using the standard Illumina protocol for amplification, hybridization, and imaging [37,64]. Beta scores were generated using Illumina’s GenomeStudio software without any normalization or background subtraction. Any value with a detection p-value above 0.01 was set to missing, and samples with more than 1.5% missing data were removed (58 samples). Non-autosomal CpGs and CpGs with greater than 10% missing data were also removed. Batch normalization was carried out using non-parametric empirical Bayes normalization using the Combat [65] function in R, using a set of 12 samples on a single array as a batch. Normalization was performed using parallel operations on random subsets of 20,000 CpGs to improve computational efficiency. Probes using Infinium I chemistry were normalized separately from those using Infinium II chemistry, and a chemistry correction was applied after normalization. The chemistry correction was based on applying a second-order polynomial fit using CpGs of differing chemistries within 50 bp of each other (correlations > 0.99 for CpG pairs within this distance) [64].

CpGs in the methylation data were filtered to include only high variation CpGs using standard deviation cutoff of 0.02. CpGs were excluded if the probe sequence mapped to multiple locations on the genome or to a different location than that listed in the annotation [37]. CpGs were also excluded if they contained SNPs in the probe with minor allele frequencies (MAF) above 0.01, as reported in dbSNP using data provided on the Illumina website. This resulted in a final set of 174,445 CpGs tested.

Genotype data were obtained using the Affymetrix Genome-Wide Human SNP array 6.0 for 717 of the 975 individuals at 906,00 genotyped and 1,622,401 imputed SNPs for a total of 2,529,001 SNPs [19,36,66]. Imputation was carried out using HapMap Phase II data with the MACH software package, Version 1.0.16 [19]. SNPs were filtered using a call rate threshold of 95% and a minor allele frequency (MAF) threshold of 0.01 within the data set. Additionally, any SNP that did not have a per-family MAF above 0.01 in at least 10% of families was removed. SNPs on haplotype specific chromosome regions or unmapped contigs were excluded due to the inability to determine distance from a CpG for these SNPs. This resulted in the final test set of 2,145,360 SNPs. DNA methylation and genotype data relevant to this study was deposited in the database of Genotypes and Phenotypes (dbGaP) with accession number phs000741, and BioProject accession PRJNA246012.

### Statistical analysis

For the analysis of this data set for the purposes of studying heritability, the family structure was modeled using a linear mixed model with a random effect for family. The software used for the primary model was the R function *lmekin* [67] in the package *coxme*, which uses a kinship matrix to model the family structure. This model [67,68] is described by the following equation:

Y=Xβ+Zb+ε

$$b∼N(0,\sigma^{2}A(\theta ))$$ (1)

$$\varepsilon ∼N(0,\sigma^{2})$$

Here, *Y* is the outcome (methylation beta score), *X* is the design matrix for fixed effects (age, gender, and the first four methylation principal components), *β* is the vector of fixed effects parameters, *Z* is the design matrix for random effects, *b* is the vector of random effects, and *ε* is the random error term. The random effect, *b*, which models the family structure, follows a normal distribution with mean 0 and variance *σ*^*2*^*A(θ)* where *θ* is an arbitrary parameter and *A(θ)* is the variance matrix of the random effect *b*. Heritability in this model is calculated using the following equation.

$$H^{2}=\frac{A(\theta )\sigma^{2}}{A(\theta )\sigma^{2}+\sigma^{2}}$$ (2)

The fixed effects covariates included in the model were age, gender, and the first four methylation principal components. Principal components were calculated using the “prcomp” function in R 2.12.1 [37,69] and were used to account for cell purity concerns and technical variation. We found that CD4^+^ cell estimates generated through our recent published method [41] were interchangeable with principal components (S6 Fig). T cell estimates were highly correlated with the first principal component with an R^2^ value of 0.83 (S6 Fig), demonstrating that including the principal components in the model adequately adjusts for cell purity in this study [37].

Highly heritable CpGs were identified based on a threshold of *H*^*2*^ > 0.4 from the model in Eq 1. Residuals were obtained for these CpGs from model 1 and tested against genotype at 2,529,001 SNPs to determine if the heritability of methylation at a particular CpG is driven by a genetic variant. A linear regression model was used to test this association with residuals produced from our model in Eq 1 versus the additive effect for genotype with Matrix eQTL in R [70]. SNPs within 5 kb of the CpG site were tested first. If no significant SNP associations (p < 10^−7^) were found, the window was expanded to 20 kb, then 100 kb, 1 Mb, 3 Mb, the entire chromosome, and finally genome-wide. Highly heritable CpGs with significant SNP associations less 1 Mb away from the location of the CpGs were classified as “cis-meQTL heritable CpGs” while those with SNP associations greater than 1 Mb or on another chromosome were classified as “trans-meQTL heritable CpGs”. CpGs with no SNP associations that met the 10^−7^ p-value threshold were classified as “genotype independent heritable CpGs”(GICs).

The *FCORR* module within the *S*.*A*.*G*.*E*. package [71] was used to calculate correlations for specific types of relative pairs. This module estimates correlation coefficient for any type of relative pair (i.e. parent/offspring or sibling/sibling) using the following equation for weighted correlation with weighted averages for subjects *x* and *y* [71,72]:

$$r_{xy}=\frac{\sum_{i=1}^{N}w_{i}(x_{i}-\bar{x})(y_{i}-\bar{y})}{\sqrt{\sum_{i=1}^{N}w_{i}{{(x}_{i}-\bar{x})}^{2}\;\sum_{i=1}^{N}w_{i}{{(y}_{i}-\bar{y})}^{2}}}$$ (3)

The weights in this equation are chosen to minimize the variance of the estimates while maintaining the proper coverage for confidence intervals [71,73]. Correlation estimates were obtained for the following main relationship types: parent-offspring, sibling-sibling, grandparent-grandchild, avuncular (aunt/uncle-niece/nephew), cousin-cousin, and mother-father (i.e. husband-wife). Correlation estimates were also obtained for the following set of gender-specific subtypes: father-son, mother-son, father-daughter, mother-daughter, brother-brother, sister-brother, and sister-sister.

Classes of CpGs were created by heritability and SNP-association (low or non-heritable (H<0.4), cis-meQTL, trans-meQTL, and GIC heritable), and these classes divided into groups by CpG island proximity as defined in Illumina’s annotation. Enrichment of classes of heritable CpGs by CpG island proximity was tested using Pearson’s Chi-Squared test. Associations among other designations were tested using Fisher’s Exact test with Monte Carlo simulation used to estimate p-values. CpGs were annotated by gene (based on Illumina’s annotation), with one observation per gene such that a CpG located near multiple genes would be listed multiple times and a CpG that is not located near any genes would not be included. Non-heritable and heritable CpGs were then classified by gene region, and other related association tests were carried out using Pearson’s Chi-Squared test.

Classes of heritable and non-heritable CpGs were then explored further with a gene ontology (GO) term analysis using Gorilla [74,75] to see if the associated genes shared any common functional characteristics. The genes in which these CpGs were located in or near (based on the Illumina annotation file) were used to conduct the GO-term analysis using a background gene list created from all CpGs on the Illumina Methyl450 array.

## Supporting Information

S1 Fig Barplot of annotated highly heritable CpGs by Roadmap chromatin states across cell types. Chromatin state legends are at right. Each group of 4 bars consist of cis-meQTLs, trans-meQTLs, GICs, and low or nonheritable CpGs from left to right.
(TIF)

S2 Fig Barplot of odd ratios (ORs) from Fisher’s tests comparing low or non-heritable CpGs versus highly heritable CpGs positioned within chromatin states associated with active chromatin or not within active chromatin. Each bar represents a different tissue or cell type. The largest ORs among primary sorted cell types (PC) are from blood (BLD; including CD3+ and CD4^+^ cells) that precede breast and adipose, and ES cells exhibit the largest ORs.
(TIF)

S3 Fig Density plots of correlation values for gender-specific family relationship types: father-son, mother-son, father-daughter, mother-daughter, brother-brother, sister-brother, and sister-sister. Densities are plotted for all 170,915 evaluated CpGs (A) and 20,163 highly heritable CpGs (B).
(TIF)

S4 Fig Beta score comparisons among sorted cells within GICs. The absolute value of deltas generated from cell type betas subtracted from T naïve betas show the greatest variation and delta values among B cells (CD19), CD14 (monocytes), granulocytes (gran), and natural killer (NK cells) (A). Hierarchical clustering of GIC-associated beta scores from sorted cell types exhibits two main clades of T cells (red) versus non-T cells (light blue) (B). Color key indicates methylation level (yellow; hypomethylated, black; 50%, blue; hypermethylated).
(TIF)

S5 Fig GICs are enriched in cell difference-related methylation values between sorted CD4^+^ T cells versus other cell types. Number of GICs exhibiting the strongest delta values (+/- 50%) between CD4^+^ T cells and cd14 (monocytes), cd19 (B cells), granulocytes (gran), and natural killer (NK) cells. The strongest deltas were not present among T cell types, and most of the differences indicate that the largest number of these CpGs are strongly hypomethylated in CD4^+^ T cells.
(TIF)

S6 Fig Boxplots of cell type estimates across all samples analyzed for heritable methylation (top left), correlation of granulocyte estimates with CD4^+^ T cell estimates (top right), and correlation of PC1 from beta scores across all individuals analyzed with CD4^+^ T cell estimates (bottom left) and granulocyte estimates (bottom right). (TIF)

S7 Fig Boxplots of twin data across highly heritable CpG categories. Boxplots of H2/h2 values across our low or non-heritable CpGs (nonherit) compared to highly heritable cis-meQTL, trans-meQTL, and GIC categories using our data (lmekin) and twin data (twin) for these same CpGs (top left). Boxplots depicting h2 values associated with genotype (h2 SNPs) from twin data (twin) across CpGs in our categories (top middle). Boxplots of unique environmental values (1-rMZ) from twin data across CpGs in our categories (top right). Boxplots of common environmental values (c2 twinACE), non additive genetic effects (d2 twin ADE) and additive genetic effects (2*rMZ-rDz) across CpGs in our categories (bottom left, bottom middle, and bottom right, respectively).
(TIF)

S1 Table Annotated DNA methylation heritability analysis results across all tested CpGs. (TXT)

S2 Table Median correlation values for gender-specific subsets of relatives for the entire set of 170,915 tested CpGs annotated according to SNP association categories. (XLSX)

S3 Table Top list of GIC regions containing 2 or more CpG sites within 10 kb of each other. (XLSX)
