# Supplementary figures and images for: Heritable DNA Methylation in CD4+ Cells among Complex Families Displays Genetic and Non-Genetic Effects
Source: PLoS One. 2016 Oct 28;11(10):e0165488. doi: 10.1371/journal.pone.0165488 (PMC5085095; doi:10.1371/journal.pone.0165488)

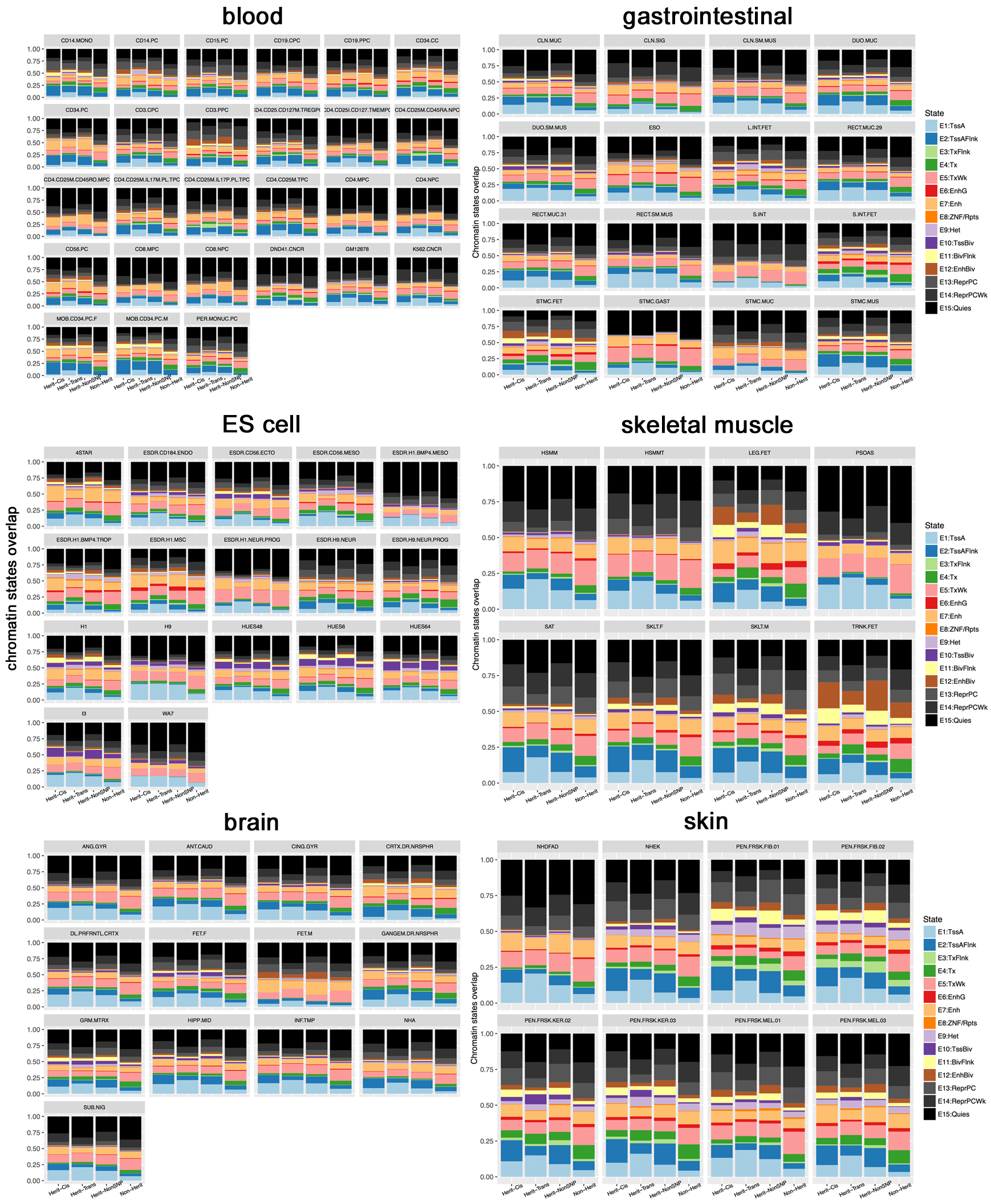

Supplement: S1 Fig — Chromatin state legends are at right. Each group of 4 bars consist of cis-meQTLs, trans-meQTLs, GICs, and low or nonheritable CpGs from left to right. (TIF) [file pone.0165488.s001.tif]

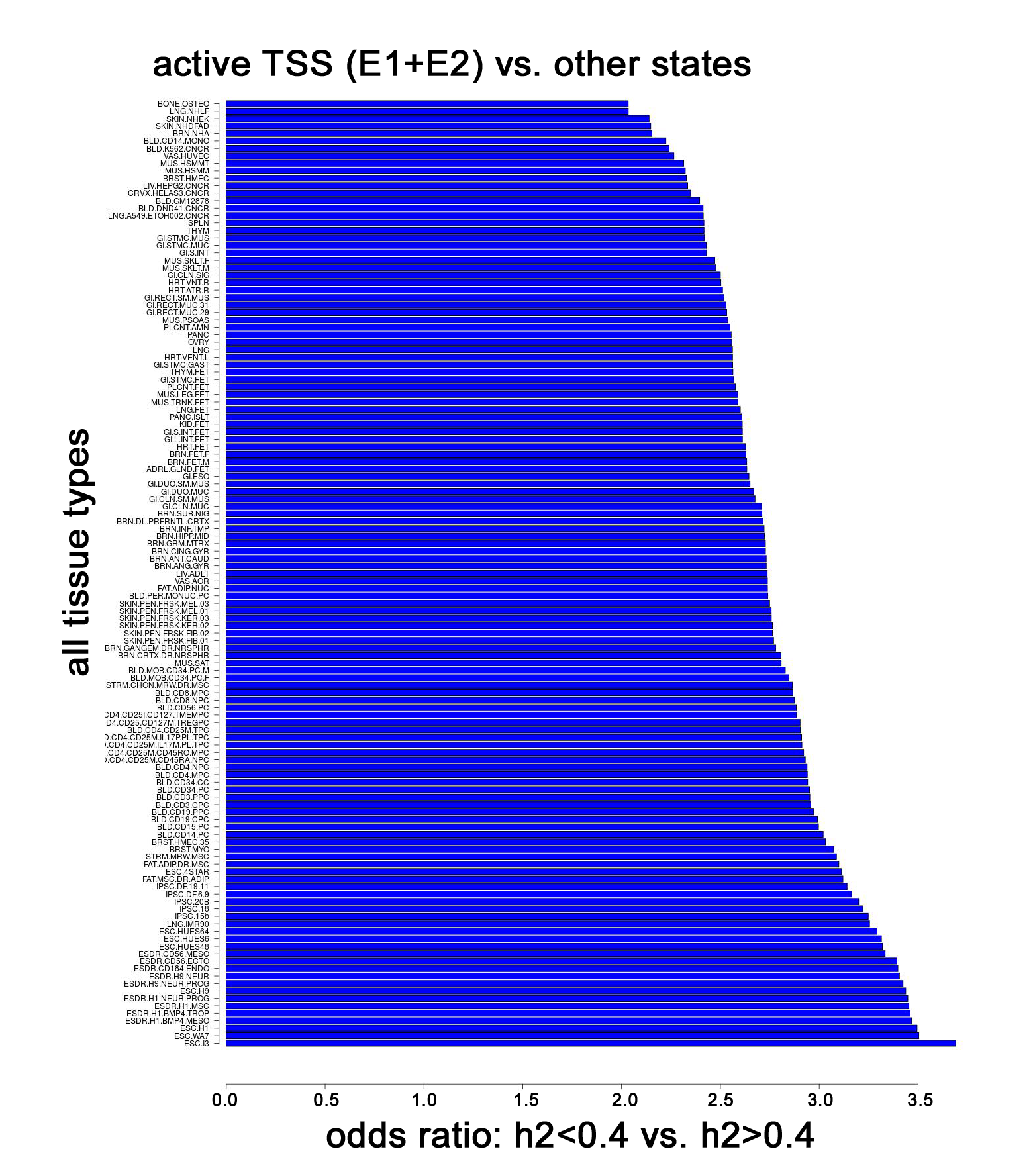

Supplement: S2 Fig — Each bar represents a different tissue or cell type. The largest ORs among primary sorted cell types (PC) are from blood (BLD; including CD3+ and CD4+ cells) that precede breast and adipose, and ES cells exhibit the largest ORs. (TIF) [file pone.0165488.s002.tif]

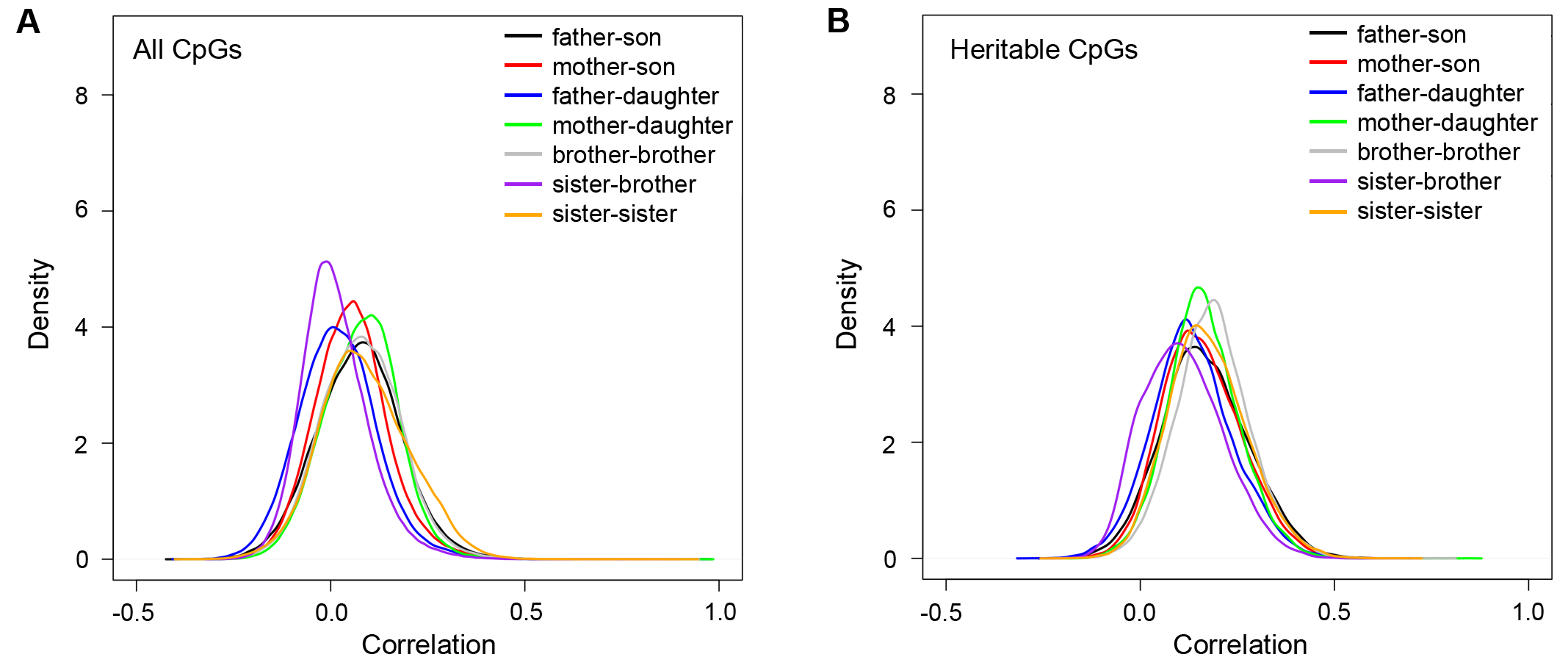

Supplement: S3 Fig — Densities are plotted for all 170,915 evaluated CpGs (A) and 20,163 highly heritable CpGs (B). (TIF) [file pone.0165488.s003.tif]

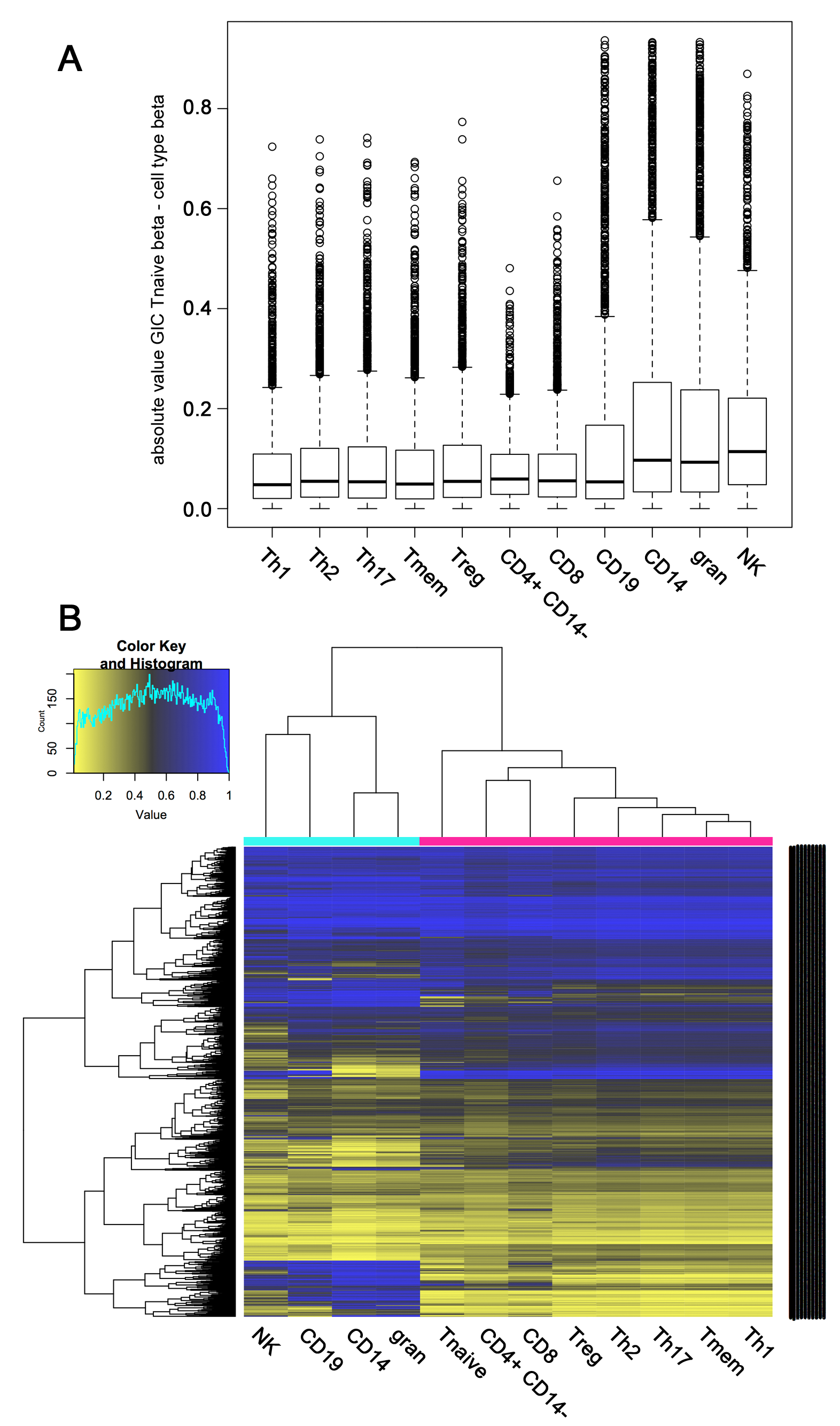

Supplement: S4 Fig — The absolute value of deltas generated from cell type betas subtracted from T naïve betas show the greatest variation and delta values among B cells (CD19), CD14 (monocytes), granulocytes (gran), and natural killer (NK cells) (A). Hierarchical clustering of GIC-associated beta scores from sorted cell types exhibits two main clades of T cells (red) versus non-T cells (light blue) (B). Color key indicates methylation level (yellow; hypomethylated, black; 50%, blue; hypermethylated). (TIF) [file pone.0165488.s004.tif]

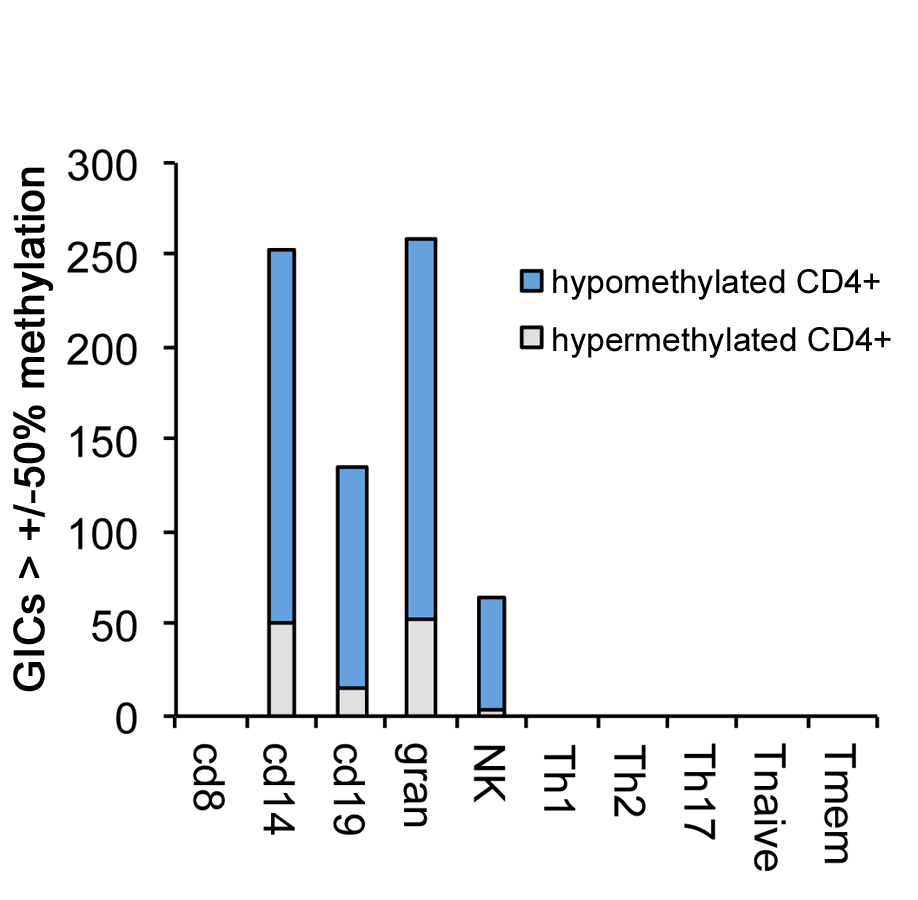

Supplement: S5 Fig — Number of GICs exhibiting the strongest delta values (+/- 50%) between CD4+ T cells and cd14 (monocytes), cd19 (B cells), granulocytes (gran), and natural killer (NK) cells. The strongest deltas were not present among T cell types, and most of the differences indicate that the largest number of these CpGs are strongly hypomethylated in CD4+ T cells. (TIF) [file pone.0165488.s005.tif]

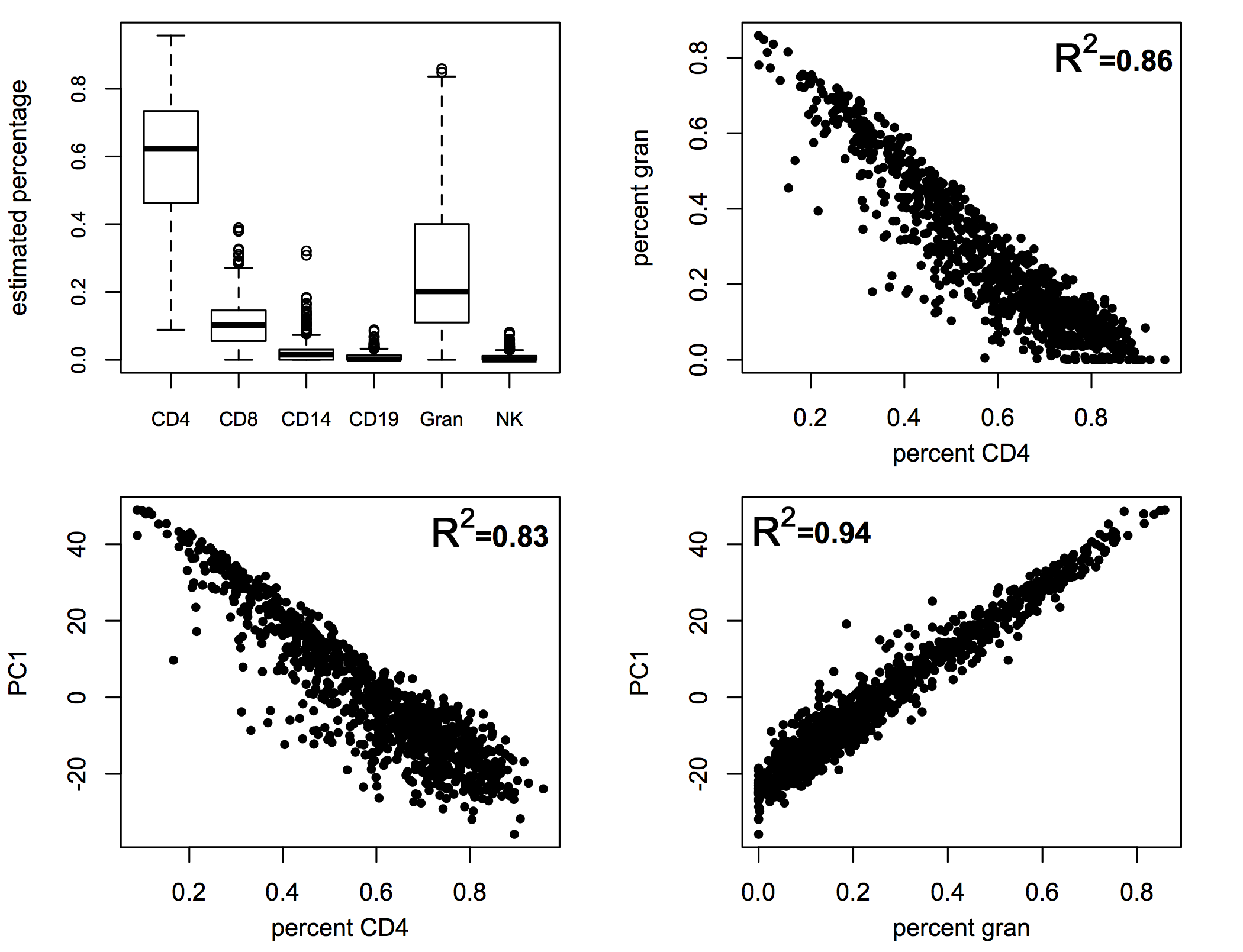

Supplement: S6 Fig — (TIF) [file pone.0165488.s006.tif]

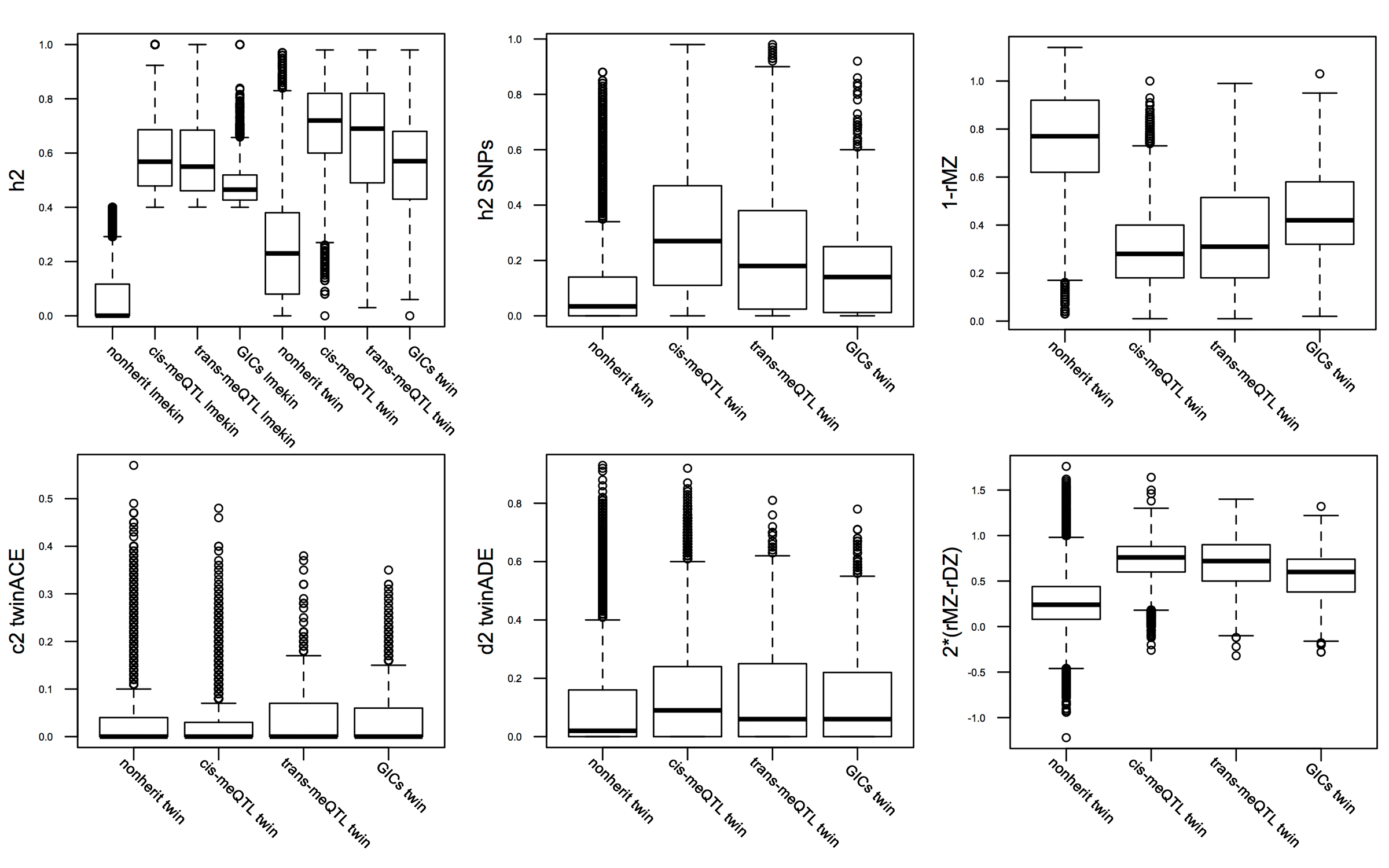

Supplement: S7 Fig — Boxplots of H2/h2 values across our low or non-heritable CpGs (nonherit) compared to highly heritable cis-meQTL, trans-meQTL, and GIC categories using our data (lmekin) and twin data (twin) for these same CpGs (top left). Boxplots depicting h2 values associated with genotype (h2 SNPs) from twin data (twin) across CpGs in our categories (top middle). Boxplots of unique environmental values (1-rMZ) from twin data across CpGs in our categories (top right). Boxplots of common environmental values (c2 twinACE), non additive genetic effects (d2 twin ADE) and additive genetic effects (2*rMZ-rDz) across CpGs in our categories (bottom left, bottom middle, and bottom right, respectively). (TIF) [file pone.0165488.s007.tif]
